# Investigating how the attributes of self-associated drug complexes influence the passive transport of molecules through biological membranes

**DOI:** 10.1016/j.ejpb.2016.03.002

**Published:** 2016-05

**Authors:** R. Inacio, D. Barlow, X. Kong, J. Keeble, S.A. Jones

**Affiliations:** King’s College London, Faculty of Life Sciences & Medicine, Institute of Pharmaceutical Science, Franklin-Wilkins Building, 150 Stamford Street, London SE1 9NH, United Kingdom

**Keywords:** Molecular aggregation, Critical aggregation concentration, Tetracaine, Chemical transport, Lag time, Skin, Percutaneous penetration, Anaesthetic

## Abstract

Relatively little is known about how drug self-association influences absorption into the human body. This study presented two hydrophobic membranes with a series of solutions containing different types of tetracaine aggregates with the aim of understanding how the attributes of supramolecular aggregate formation influenced passive membrane transport. The data showed that aqueous solutions of the unprotonated form of tetracaine displayed a significantly higher (*p* < 0.05) passive membrane transport compared to solutions with mixtures of the unprotonated and protonated drug microspecies (e.g. transport through the skin was 0.96 ± 0.31 μg cm^−2^ min^−1^ and 1.59 ± 0.26 μg cm^−2^ min^−1^ respectively). However, despite an enhanced rate of drug transport and a better membrane partitioning the unionised molecules showed a significantly longer (*p* < 0.05) lag time to membrane penetration compared solutions rich in the ionised microspecies. Analytical characterisation of the solutions applied to the apical surface of the membranes in the transport studies showed that larger tetracaine aggregates with smaller surface charge gave rise to the longer lag times. These large aggregates demonstrated more extensive intermolecular bonding and therefore, it was suggest that it was the enhanced propensity of the unionised species to form tightly bound drug aggregates that caused the delay in the membrane penetration.

## Graphical abstract

**Figure:**
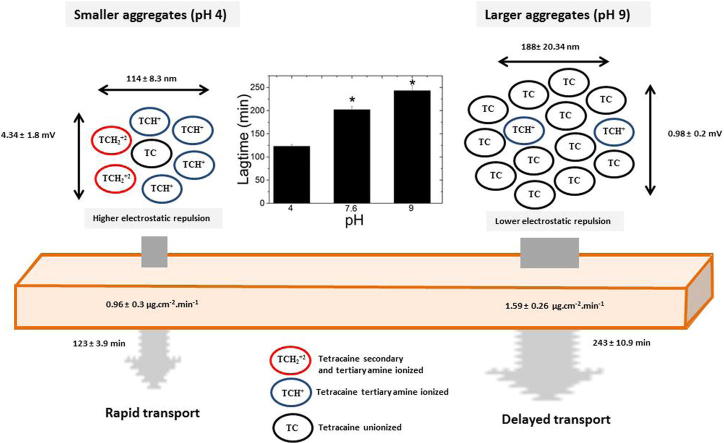

## 1 Introduction

Pharmacologically active compounds can display amphiphilic properties and this can lead to molecular aggregation in solution. Aggregate formation can have a significant impact on the biological function of a molecule because it can influence the passage through membranes. These changes are driven by alterations in diffusion speed and modification of the interactions with other molecules [1], [2], [3]. However, the physical interactions between the aggregated and non-aggregated entities, the multiple routes that molecules can take through a barrier and the potential for both the unaggregated and aggregated drug to pass through a membrane mean that the influence of molecular aggregation upon transport is a complex field of study that warrants further investigation.

One example of a molecule that is known to aggregate in solution is tetracaine [4]. Its self-association is thought to be driven by intermolecular tertiary amine hydrogen bonding and amine–ester hydrogen bonding [4]. Tetracaine has a good bioavailability when applied topically to biological membranes [5], [6], [7], but it can have a slow onset of topical anaesthesia (30–60 min), which hinders its effective clinical use in certain contexts [8], [9], [10]. Attempts to enhance tetracaine diffusion across the membranes have been reported in the literature [11], [12], [13], but there has been very little investigation into how molecular aggregation influences the passive diffusion of this drug and thus the consequences of molecular aggregation on its speed of onset remain unknown.

Tetracaine is commercially available as a 4% gel (formulation pH ∼ 9) and can exist in solution as three different microspecies depending on the pH. Tetracaine base (TC) is thought to be the efficient in crossing biological barriers [14], and it is the dominant microspecies above pH 9. Below pH 6.5 both the tertiary (TCH^+^) and secondary amine (TCH_2_^2+^) are protonated and the microspecies TCH^+^ prevails at the physiological pH of the skin (4.2–6.5). It is generally accepted that the tetracaine tertiary amine binds to the sodium channels, blocking the sodium influx, inhibiting nerve cell depolarization and preventing the propagation of nerve cell impulses [15], but which is the most effective species to gain rapid penetration into the skin remains less clear.

The aim of this work was to use tetracaine as a model drug in order to gain a better understanding of how the physical attributes of supramolecular masses formed as a consequence of drug aggregation influence hydrophobic membrane transport. Tetracaine was chosen as the model agent due to the fact that its amphiphilic characteristics could be manipulated by simply altering the pH of aqueous solutions containing the drug when it was above its critical aggregation concentration (CAC). This allowed a series of aqueous vehicles where the degree of ionization of both the tertiary and secondary amine was varied to be presented to two hydrophobic membranes, porcine skin and silicone, in the anticipation that different types of drug aggregates and different transport rates could be recorded and analysed. Skin was chosen as an example of a biological barrier as there was evidence in the literature that tetracaine could pass through this membrane [5], [6], [7]. Photon correlation spectroscopy was employed to determine the critical aggregation concentration at which nanosized aggregates were formed and zeta potential measurements provided details of the electrostatic interactions. Fourier transform infrared spectroscopy (FTIR) and ^1^H NMR were employed to investigate the molecular arrangement and intermolecular bonding between the different microspecies in solution.

## 2 Materials and methods

### 2.1 Materials

Acetonitrile and methanol both HPLC grade, grade A glass pipettes, clear glass high performance liquid chromatography (HPLC) vials with crimpable lids and 0.45 μm nylon filter papers were purchased from Fischer Scientific (Leicester, UK). Tetracaine base BP grade (99.9%) and deuterium oxide (99.9 atom%) were supplied by Sigma Aldrich (Dorset, UK). Concentrated hydrochloric acid and sodium hydroxide were from Fluka (Dorset, UK). Sodium acetate, potassium dihydrogen phosphate and 1-Octanol were provided by Alfa Aesar (Heysham, UK). Silicone membranes with a thickness of 0.25 mm were purchased from GBUK Healthcare (Selby, UK).

### 2.2 Tetracaine p*K*a determination

The automatic titration system used in this study comprises an autoburette (Dosimat 765 liter ml syringe, Metrohm, Buckingham, UK) and pH meter (MP230 Mettler Toledo, Leicester, UK) with a pH electrode (Metrohm, Buckingham, UK). A 0.1 M KCl electrolyte solution was used to maintain the ionic strength. The temperature of the test solutions was maintained in a thermostatic jacketed titration vessel at 25 °C ± 0.1 °C by using a temperature controller (Techne TE-8J, Sigma–Aldrich, Dorset, UK). The solution under investigation was stirred vigorously during the experiment. A pump with speed capability of 20 mL min^−1^ (Mini-plus, Gilson, Luton, UK) was used to circulate the test solution through a quartz flow cuvette using a cuvette with a path length of 0.1 cm. The flow cuvette was mounted on an UV–visible spectrophotometer (HP 8453, Agilent, Cheadle, UK). All instruments were interfaced to a computer and controlled by a Visual Basic program. Automatic titration and spectral scans adopted the following strategy: the pH of a solution was increased by 0.1 pH unit by the addition of KOH from the autoburette; when pH readings varied by <0.001 pH unit over a 3 s period the spectrum of the solution was then recorded. The cycle was repeated automatically until the defined end point pH value was achieved. All the titration data were analysed with the pHab program [16]. The microspecies plot was generated with the HYSS program [17].

### 2.3 Tetracaine aggregate analysis

#### 2.3.1 Photon correlation spectroscopy characterisation

Changes in derived count rate and zeta potential of the donor solutions were tracked using photon correlation spectroscopy (PCS) (Malvern Nanoseries Zetasizer, Malvern Instruments Ltd., Malvern, UK). Measurements were taken at a scattering angle of 173°. Refractive index and viscosity constants were set at 1.33 and 0.88 mPa s, respectively. Samples were filtered through a 0.45 μm cellulose nitrate filter prior to the analysis. The scattering information was determined at increasing tetracaine molar concentrations in acetate buffer (0.1 M) at pH 4, 6, 7.6, 9 and 10. The critical aggregation concentration was determined from the data discontinuity in the linear model applied to the derived count rate data; this was confirmed by the application of a second derivative function (OriginPro 9.1 Software, OriginLab, Northampton, USA). The size of the molecular aggregates was detected by converting the signal into a hydrodynamic radius using the Stokes–Einstein equation given in (Eq. (1)), where *k* is the Boltzmann constant, *T* is the absolute temperature and *η* is the solvent viscosity. The size of the molecular aggregates was determined above critical aggregation concentration ca. 87–95% of tetracaine saturation in each aqueous vehicle at pH 4, 6, 7.6, 9 and 10. The pH was adjusted to the required value when necessary by adding NaOH (1 M) or acetic acid. Zeta potential magnitude was determined at pH 4, 7.6 and 9 using the same solutions described above.

(1) $$R_{H}=\frac{\mathrm{kT}}{6\pi \eta D}$$

#### 2.3.2 Molecular dynamic studies

Tetracaine self-assembly was generated from the crystal structure reported by Nowell et al. [18]. The atomic co-ordinates and unit cell parameters were obtained from the Cambridge Structural Database; entry XISVOK [19]. The assembly was generated (2 × 2 × 2 unit cells) using the Mercury software [20] and then visualised using Accelrys Viewerlite v5.0 (Biovia, San Diego, USA).

#### 2.3.3 Apparent distribution coefficient

The apparent drug distribution coefficients were measured using tetracaine saturated solutions at room temperature as previously described, using n-octanol in acetate buffer (0.1 M) at pH 4, 6, 7.6, 9 and 10 [21]. After phase separation the aqueous phase was withdrawn and samples were centrifuged at 13,000 rpm (Biofuge, Heraeus, Germany) and aliquots of the liquid phase were then transferred into vials. The samples were analysed using HPLC. The apparent distribution coefficient (*D*) was defined as the ratio of the drug concentration in octanol (*C*_0_) to the total concentration of ionised (*C_i_*) and unionised (*C_u_*) drug in the aqueous phase (Eq. (2)).

(2) $$D=\frac{C_{0}}{(C_{i}+C_{u})w}$$

#### 2.3.4 Fourier transform infrared spectroscopy (FTIR) characterisation

Tetracaine solutions at pH 4, 6, 7.6, 9 and 10 were prepared in deuterium oxide at the same concentrations used for molecular aggregate size analysis. Deuterium oxide (D_2_O) was employed in the solutions as it dampened the solvent signal in the 1700–1300 cm^−1^ range. The pH was adjusted with NaOH (1 M) or acetic acid. The samples were loaded into a demountable universal transmission cell system (Omni-Cell, Specac Ltd., Kent, UK) fitted with CaF_2_ windows and a 25 μm mylar spacer (Specac Ltd., Kent, UK). The infrared spectra were recorded from 4500 to 1000 cm^−1^ using a Spectrum One spectrometer (Perkin Elmer Ltd., Bucks, UK) and spectral analysis was performed with Spectrum software version 5.3.1 (Perkin Elmer Ltd., Bucks, UK). After normalisation of transmittance, background subtraction and baseline correction of the spectra, hydrogen bonding interactions in the carbonyl group region were determined by analysing spectral shifts of the C=O stretching peak as described previously [22].

#### 2.3.5 ^1^H NMR spectroscopy

Tetracaine solutions at pH 4, 6, 7.6, 9 and 10 were prepared in deuterium oxide at the same concentrations used for molecular aggregate size analysis. The spectra of each solution were obtained with a Bruker Avance DRX400 NMR spectrometer (Bruker, Coventry, UK). A 600 μL aliquot of each sample was used for the measurements which were conducted at 400 MHz with 5000 scans. The peak assignments in the spectra were made and supported using the predicted spectra (ChemNMR software, Perkin Elmer, Beaconsfield, UK).

### 2.4 Transport studies

To eliminate any thermodynamic influence on the drug passive transport across both membranes, tetracaine was formulated as a saturated solution i.e. at a thermodynamic activity of unity [23], [24]. Using a fine suspension to produce the saturated solutions allowed visual inspection to confirm saturated conditions were maintained throughout the experiments. The individually saturated solutions at pH 4, 6, 7.6, 9 and 10 were prepared by adding excess tetracaine to acetate buffer (0.1 M). The pH of each solution was adjusted to the required value when necessary by adding NaOH (0.1 M).

Adult pig ears were obtained from a local abattoir. The ears were removed from the carcass after hair removal. Any ears that were obviously damaged were discarded. The ears were cleaned with water, the residual water on the skin surface was immediately removed by blotting with tissue, visible residual hairs were trimmed carefully and the ears were stored at −20 °C (no more than three months before using). Porcine skin was defrosted and the subcutaneous fat carefully removed using a scalpel. Both the silicone membrane (0.25 mm thick, used as obtained) and the porcine skin were cut into pieces of a suitable size and mounted in the Franz diffusion cell (University of Southampton, UK) [25] with surface areas of approximately 2 ± 0.2 cm^2^ and receiver compartment volumes of approximately 9.5 mL. Porcine skin was mounted with the *stratum corneum* facing the donor compartment. The receptor compartment of the cells was filled with acetate or phosphate buffer (0.1 M) at the same pH as the donor compartment. To ensure there was no leakage, the cells were inverted and visually checked. Leaking cells were excluded from the experiments. In order to guarantee uniform mixing of the receptor phase and maintenance of sink conditions (drug concentration in the receiver fluid did not exceed 10% of its saturated solubility), 13 mm magnetic stirrer fleas were placed in the receptor compartment of the Franz cells. Five cells were used for each experiment and they were allowed to equilibrate for 30 min prior to use by immersing the receptor compartments in a 37 °C water bath (Grant instruments, Cambridge, UK). A 3 mL sample of each saturated solution was applied to the apical surface of the membrane to initiate the transport studies. At specified time intervals over a period of 6 h and 22 h (for the silicone and porcine membrane respectively), 1 mL samples were taken out of sampling arm of the receiver compartment and immediately replaced by fresh acetate buffer of equal volume and temperature. Samples were stored at room temperature until HPLC analysis was carried out. Cumulative amounts of drug (μg) penetrating the membrane per unit diffusional surface area (cm^2^) were corrected for previous sample removal and plotted against time (min). The linear region of the plot (*R*^2^ > 0.99 over at least 5 points) was defined as the steady-state flux. Membrane thickness was measured before and after the transport experiments using a Vernier micrometer (No. 436.1 Series 0–25 mm) purchased from Starrett (Jedburgh, UK), in order to check for swelling which would indicate vehicle–matrix interaction [26]. Lag time was determined by the intercept of the line extrapolated from the linear model applied to the data in order to calculate steady-state flux with the x axis and it was provided in min.

### 2.5 Tetracaine HPLC assay

A liquid chromatography pump (P680 HPLC pump, Dionex, Surrey, UK) with an ASI-100 automated sample injector (Dionex, Surrey, UK) connected to a PDA-100 photodiode array detector (Dionex, Surrey, UK), was used for the quantitative determination of tetracaine. The HPLC system was connected to a computer with a Chromeleon software (Dionex, Surrey, UK), which was used to record and analyse the chromatograms. A Luna C18 (5 μm, 250 × 4.6 mm) column (Phenomenex, Cheshire, UK) was used with a 50:25:25 acetate buffer (0.1 M)/methanol/acetonitrile mobile phase at pH 4 and a flow rate of 1 mL min^−1^. Volumes of 50 μL were injected onto the column and tetracaine was analysed at a wavelength of 311 nm. The method was previously shown to be fit for purpose in terms of linearity (*R*^2^ > 0.999), peak symmetry (0.8–1.3) and sensitivity (the limit of detection was 3.3 μg mL^−1^ and the limit of quantification was 9.7 μg mL^−1^) in accordance with the limits described by the International Conference on Harmonisation guidelines [27]. The tetracaine retention time was 7.62 min. The liquid chromatography method was found to be stability indicating and was able to detect tetracaine hydrolytic degradation products p-n-butylaminobenzoic acid and diethylaminoethanol [28].

### 2.6 Statistical data analysis

Statistical evaluation was carried out using a statistical package for social sciences software (SPSS® version 11.0, SPSS Inc., Chicago, IL, USA). The Kolmogorov–Smirnov test was used to check the normality of the data. Data were analysed by one-way ANOVA and post hoc comparisons of the means of individual groups were performed using Tukey’s Honestly Significant Difference test. In all cases, a statistically significant difference was defined as when *p* ⩽ 0.05. All values were expressed as mean ± standard deviation. The number of replicates was 5 in the permeation studies and 3 in the apparent distribution coefficient determination and light scattering studies.

## 3 Results and discussion

### 3.1 Tetracaine p*K*a determination

**Fig. 1:**
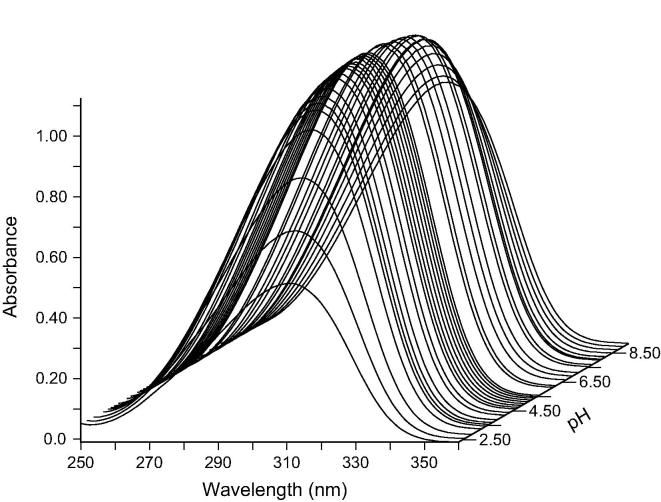
UV-spectrum of tetracaine (560 μM) between pH 2.36 and pH 10.94 starting in 20.1 mL of KCL (0.1 M) at 25 °C using a cuvette with a path length of 0.1 cm. Total points represented are 44.

The analysis of the titration data using the pHab program revealed two inflection points at pH values of 2.48 ± 0.03 and 8.56 ± 0.02 in the plots of tetracaine solution UV absorbance versus its pH and these were considered to be the approximate values of tetracaine p*K*a’s (Fig. 1). The p*K*a value at 2.48 ± 0.03 was assigned to the protonation on nitrogen of the tertiary amine and the p*K*a value of 8.56 ± 0.02 was assigned to the secondary amine. There was a good agreement between the experimentally determined values in this work and those described in the literature. For example, Iglesias et al., (2012) reported a tetracaine p*K*a value of 3.41 and 8.24 [15]. Therefore, the percentage of each microspecies in solution (TC, TCH^+^ and TCH_2_^2+^) at increasing pH was calculated using the experimentally determined p*K*a’s from this work (Table S1, Supplementary data). The commercial topical product displayed an apparent pH of ca. 9 and therefore it was considered to present tetracaine predominantly as a unionised molecule (TC) when it was used clinically to induce topical anaesthesia.

### 3.2 Tetracaine supramolecular aggregate analysis

#### 3.2.1 Photon correlation spectroscopy characterisation

**Fig. 2:**
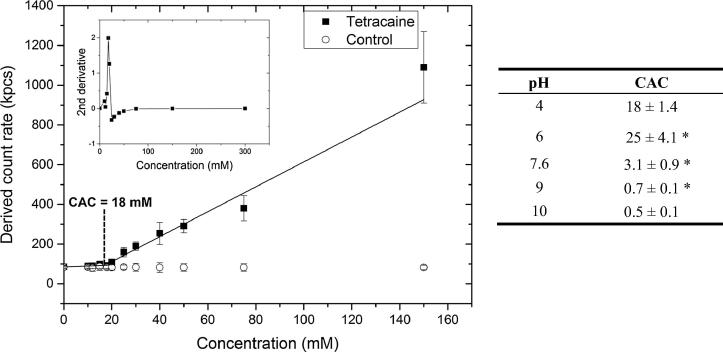
Graph depicting the changes in total light scattering for samples at pH 4. Inset graph represents the application of a second derivative function that determined the discontinuity in the slope of the derived count rate data. Critical aggregation concentration (CAC) values for each test system are displayed in the table. ^∗^*p* < 0.05 represents significant difference between CAC results (Tukey’s Honestly Significant Difference test). Each point represents mean ± standard deviation (*n* = 3).

**Fig. 3:**
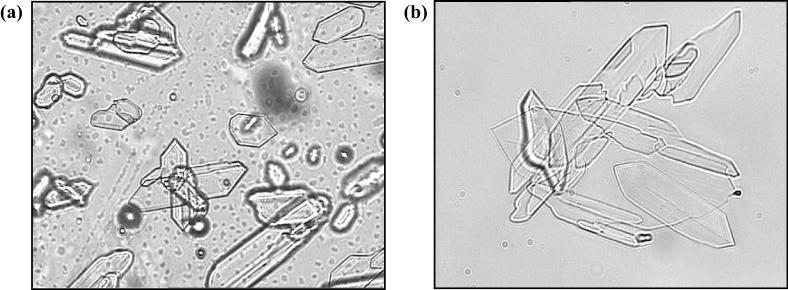
Light microscopy (Olympus BX50F, Japan) at a magnification of 40× (a) tetracaine commercial product and (b) tetracaine test system at pH 9 above critical aggregation concentration.

The critical aggregation concentration of the drug solutions was significantly higher (*p* < 0.05) when they contained species with some proportion of both the ionised tertiary and secondary amines, i.e. the critical aggregation concentration was 18 ± 1.4 mM (Fig. 2) at pH 4 and 3 ± 0.9 mM at pH 7.6 compared to 0.7 ± 0.1 mM at pH 9 and 0.5 ± 0.1 mM at pH 10. The two CAC values recorded at pH 9 and 10 were not significantly different (*p* > 0.05). This trend in the data indicated that the self-association process was more favourable when the unionised tetracaine microspecies were prevalent in aqueous solutions (for additional information see Fig. S1 and Table S1 included in the Supplementary data). The 4% (w/w) tetracaine commercial gel presented the molecule to the skin as a two phase system, and microscopy analysis showed that it contained drug microcrystals (Fig.3a and b). In addition, the CAC data indicated the active molecules in the bulk solution of this formulation were probably in both aggregated and unaggregated states. Together these physical data demonstrated that the commercial system was a complex delivery system.

**Fig. 4:**
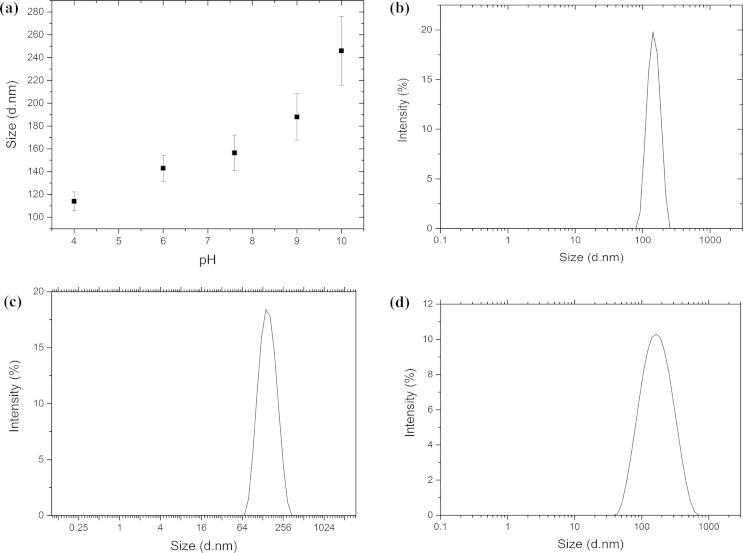
Graph depicting the changes in (a) molecular aggregates size (nm) at increasing pH of the donor solution and size distribution above critical aggregation concentration, (b) at pH 4, (c) at pH 7.6 and (d) at pH 9. Each point represents mean ± standard deviation (*n* = 3).

**Fig. 5:**
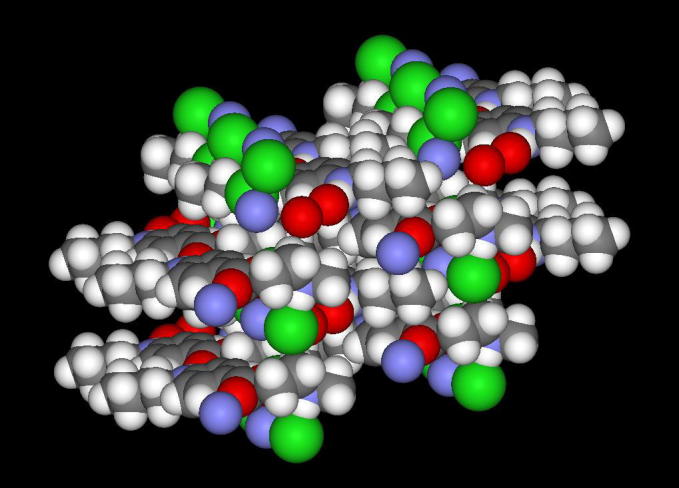
Screenshot of a 16 molecule tetracaine·HCl self-assembly generated from the crystal structure using the Mercury software and visualised using Accelrys Viewerlite v5.0. Atom colour scheme: H = white, C = grey, O = red, N = blue, Cl = green. (For interpretation of the references to colour in this figure legend, the reader is referred to the web version of this article.)

The relatively high polydispersity index (⩾0.25) measured for all test systems indicated that there were different sized aggregates in solution and this suggested that the aggregates did not form a highly ordered structure such as a micelle (Fig. 4b–d). The size of the aggregates present in solution above the tetracaine CAC was shown to be significantly greater (*p* < 0.05) when the proportion of charged species in solution was reduced, i.e., the average aggregate size was 114 ± 8.3 nm at pH 4 whilst it was 188 ± 20.4 nm at pH 9 (Fig. 4a). The zeta potential of the solutions containing more of the charged amine species was also significantly higher (*p* < 0.05) (4.34 ± 1.8 mV at pH 4 vs 0.98 ± 0.2 mV at pH 9). The zeta potential data suggested that the supramolecular structures formed from the ionised tetracaine microspecies were influencing the surface charge of the aggregate. Molecular modelling of the tetracaine aggregate structure using the crystallographic data supported this hypothesis. When tetracaine forms crystals the charged amine is most likely to orientate towards the surface of the structure rather than the interior, and if this also occurred in the liquid state then this would be the reason why the aggregate charge was altered by the proportion of ionised species in solution (Fig. 5). Whilst it is accepted that the supramolecular formation in solution is not the same process as supramolecular structure formation during crystallisation this explanation provides a hypothesis for further studies to test.

#### 3.2.2 Tetracaine partitioning

**Fig. 6:**
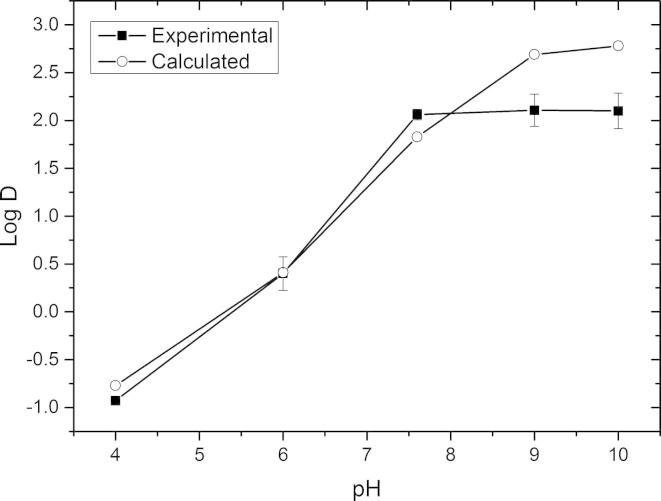
Experimental and calculated distribution coefficient values for tetracaine at different pHs. Each point represents mean ± standard deviation for the experimental data (*n* = 5).

The experimentally determined Log *D* values of tetracaine donor solutions at pH 4.0, 6.0, 7.6, 9 and 10 showed the same trend as the calculated values (Fig. 6). The most significant differences were surprisingly at the higher pH’s of 9 and 10 where the experimental distribution coefficients were recorded as 2.1 ± 0.16 and 2.01 ± 0.18 respectively. As the amount of tetracaine detected in the aqueous phase of the partitioning experiments was above the experimentally determined critical aggregation concentration for all test systems it was assumed that the differences in the predicted and measured values were a consequence of supramolecular tetracaine structures. As the experimental partition coefficient was lower than the predicted value this indicated that molecular aggregates were in fact more hydrophilic than the unaggregated tetracaine. This was a surprising result, but it was possible that the aggregates did contain a component of the ionised amine even at pH 9 and 10, and if, as the molecular simulation images suggest, these are orientated more preferably towards the surface of the aggregate it is possible that these ionised species increased the hydrophilicity of aggregates compared to the unaggregated molecules that were assumed to exist in the calcaulated values.

#### 3.2.3 Fourier transform infrared spectroscopy

**Fig. 7:**
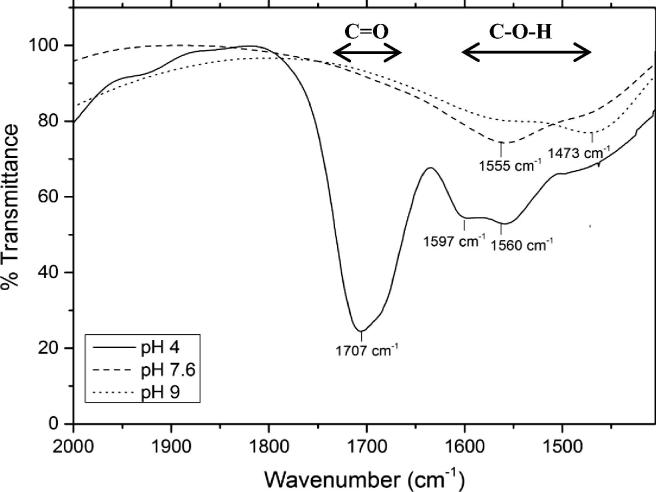
Fourier transform infrared (FTIR) transmittance spectra (arbitrary units) of the C=O band of tetracaine in deuterium oxide at pH 4, 7.6 and 9 above the experimentally determined critical aggregation concentration.

The FTIR spectra of tetracaine at pH 4 were characterised by the presence of the C=O group (1707 cm^−1^) and two vibrational bands at 1597 cm^−1^ and 1560 cm^−1^, which fell within the hydroxyl group absorption frequency (Fig. 7). The hydroxyl bands were thought to arise due to the intermolecular interactions of tetracaine in solution [29], [30]. Similar effects have previously been observed with other drug–drug interactions in solution [31]. Above pH 4, the stretching vibrational band assigned to the C=O group was no longer present (Fig. 7, data at pH 6 and 10 are not shown for greater visual clarity), and there was a significant upfield shift and broadening of the vibrational bands assigned to the hydroxyl group (e.g. 1473 cm^−1^ at pH 9). This suggested that there was an increasingly favourable environment for aggregation driven by stronger intermolecular interactions in the solutions at a higher pH. The FTIR data suggested that the tetracaine molecules responsible for the supramolecular structure formation utilised hydrogen bonds formed through an N—H—O=C association. It was still considered a possibility that N—H—N—H association could play a role in the aggregation process [4], but because the IR signals from the amines in water were very weak it was impossible to arrive at firm conclusions with regard to the amine involvement in the tetracaine–tetracaine association.

#### 3.2.4 ^1^H NMR spectroscopy studies

**Fig. 8:**
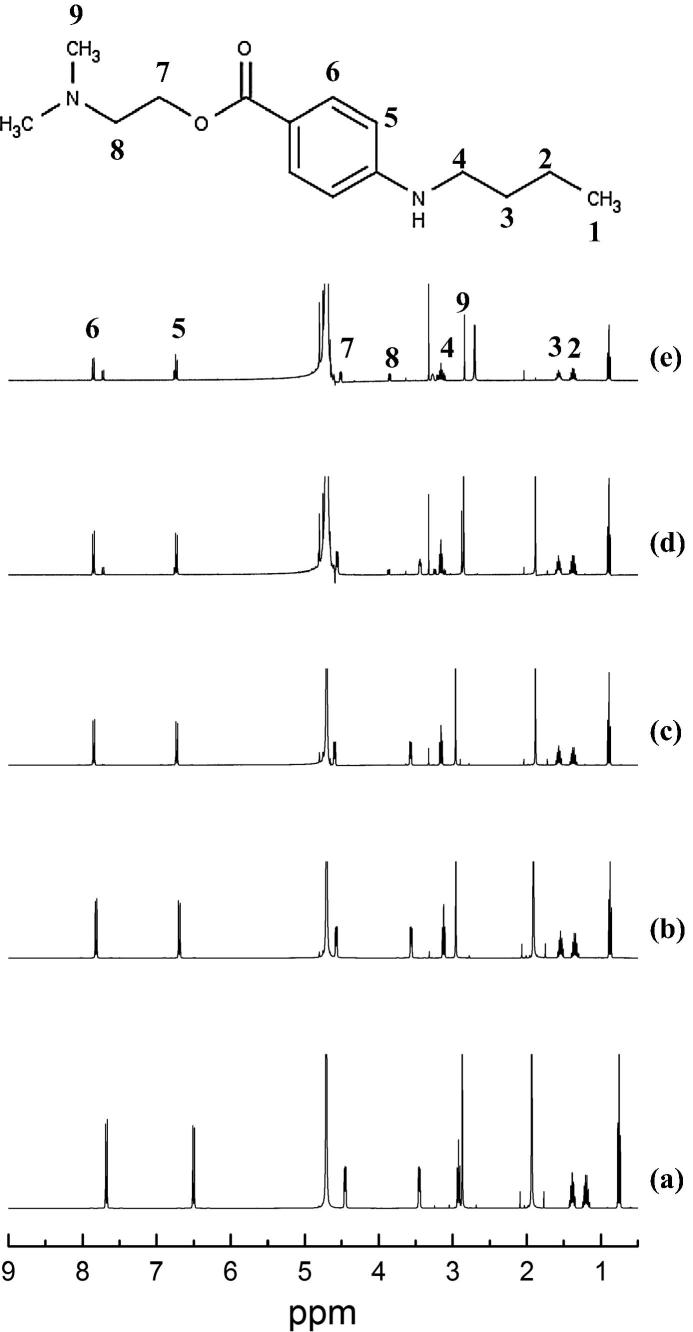
^1^H NMR spectra of tetracaine in deuterium oxide, (a) pH 4, (b) pH 6, (c) pH 7.6, (d) pH 9 and (e) pH 10 in a concentration above the experimentally determined critical aggregation concentration.

There were 9 protons that gave clearly resolved peaks in NMR spectra (Fig. 8, Table S2). The resonance of the methylene protons H1, H2, H3 of the alkyl chain of the secondary amine and the methyl group (H9) attached to the tertiary amine remained essentially unchanged regardless of the pH environment of tetracaine. However, the chemical shift of all the other protons tended to move downfield (deshielding effect) with an increasing pH. The methylene protons of the alkyl chain adjacent to the oxygen of the ester group (H7) displayed the largest frequency change of ∼1.16 ppm as the pH increased from 4 to 10. In addition, the H7 proton changed in its multiplicity appearing as a quartet at pH 9. The other significant proton chemical shift change was the methylene adjacent to the tertiary amine (H8) which moved ∼0.4 ppm as the pH increased from 4 to 10. The chemical shifts of the aromatic protons attached to the benzene ring and the methylene protons adjacent to the secondary amine changed by relatively small amounts, but there was a change in the multiplicity of these protons at pH 10.

The decrease in the nuclear shielding as the vehicle pH increased could indicate a possible change in the free movement of the tetracaine molecules in solution, suggesting that the rotation of the methylene protons of the lateral alkyl chains was inhibited by intermolecular bonding between tetracaine molecules [32]. This increasing steric hindrance, which increased with pH, indicated, in a similar manner to the FTIR data, that when more of the non-ionised tetracaine molecules were present in solution a more tightly bound supramolecular structured was formed. Previous studies on the interactions of model polyamines in supramolecular structures have shown similar results [33], [34].

### 3.3 Tetracaine transport studies

Two membranes were used in this work, a synthetic silicone membrane, which presents a homogeneous non-porous barrier to diffusion, and porcine skin, which is the typical *in vivo* preclinical model used to characterise topically applied medical products (due to its similar permeability and histological characteristics to human skin [35], [36]). Porcine skin allows molecules to pass through its barrier both via the confluent structure presented by the cells in the various skin layers and the pores introduced into the skin structure via hair follicles and sweat glands. In contrast, the synthetic silicone membrane only allows transport through its barrier via the classical partitioning and diffusion process which has been modelled by Higuchi [37]. By combining the data from a series of test systems using both membranes it was hoped that an understanding of how the molecules passed through the barriers could be obtained.

**Fig. 9:**
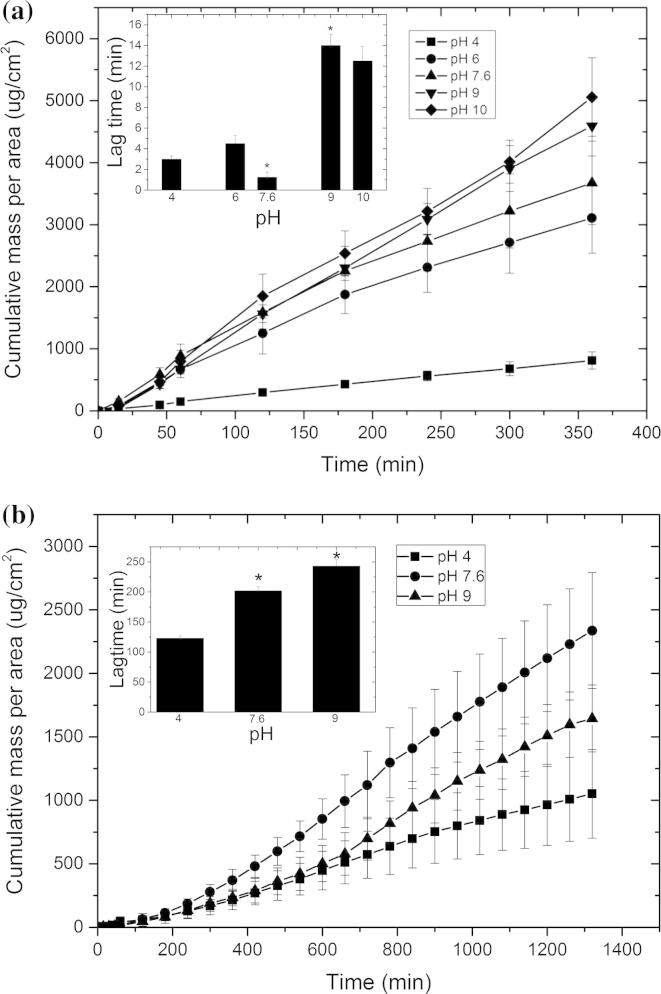
Tetracaine transport profile through (a) silicone membrane using an infinite dose of aggregated drug at pH 4, pH 6, pH 7.6, pH 9 and pH 10 and through (b) porcine skin using an infinite dose of aggregated drug at pH 4, pH 7.6 and pH 9. The inset graph represents lag time versus pH. Each point represents mean ± standard deviation (*n* = 5). ^∗^*p* < 0.05 (one-way ANOVA with Tukey’s HSD test).

The data confirmed that at each pH the synthetic membrane (Fig. 9a) and porcine skin (Fig. 9b) were acting as rate limiting barriers. The experiments applied an infinite dose of the drug that was saturated in an aqueous solution which was thought not to interact with the membrane. A calculation of the total drug transport through the membrane in each experiment suggested that no drug depletion took place and hence it was assumed that tetracaine was constantly supplied in the transport studies at unity to the barrier. Sink conditions in the receiver compartment were maintained for each experiment, i.e., tetracaine concentration in the receiver fluid did not exceed 10% of the saturated solubility at each experimental pH [38]. Therefore, it was assumed that the cumulative rate of drug passing the membrane taken from the exponential portion of the cumulative drug concentration vs time plots could be considered to be the steady-state flux.

The transport data showed in general that the drug flux and penetration lag time increased as the solution pH of the donor solutions was increased (Tables S3 and S4). This increase in drug flux was thought to be a consequence of the increase in the percentage of unionised tetracaine in the donor solution, which had a greater affinity for the membrane. This hypothesis was built from the distribution data and the fact that this trend was shown in both membranes suggested that the main mechanism of transport was passive diffusion through the confluent barrier presented by the cells of the skin rather than transport via the follicular route (Fig. 9).

Traditionally, when the transport of a non-aggregated drug system increases the membrane penetration lag time decreases. However, in the present study the lag time increased. For example, when the unionised fraction increased in the donor solution increased from 0.03% to 73.4% (pH 4 and pH 9 respectively) there was a significant increase (*p* < 0.05) in lag time from 3.1 ± 0.8 min to 14 ± 1.1 min and from 123 ± 3.9 to 243 ± 10.9 min using the synthetic membrane and porcine skin respectively (Tables S3 and S4). These data were thought to be a consequence of the supramolecular structuring of tetracaine having a significant functional effect on the drug transport kinetics. The two most likely causes of the increases in lag time were considered to be specific interactions between the drug and the barrier and the different availabilities of membrane transportable species in the donor solutions [39]. However, as the aggregate characterisation data had already shown that larger, more tightly formed drug supramolecular complexes were formed at high pH’s it was thought that the greater restriction of the rapidly diffusing unionised microspecies of tetracaine by the large hydrophobic masses, which may themselves have difficulty in passing through or leaving the barrier, was the main cause of the general trend of an increased lag time as pH of the donor solutions increased [40].

There was an interesting exception in the general trend of an increase in membrane penetration lag time as the donor solution pH was increased, i.e., when tetracaine showed the lowest penetration lag time at pH 7.6 through the synthetic membrane. This effect was not mirrored in the porcine skin. However, as the light scattering data, the partitioning data and the NMR data did not show anything particular in the tetracaine solutions at pH 7.6 and the reason for this effect could not be derived from the current data set. It was assumed that at pH 7.6 the self-assembled structure of tetracaine was generated in such a manner that it provided optimal conditions for the tetracaine to pass through the silicone membrane. There was a discrepancy in the skin and silicone membrane data regarding the ability of tetracaine to pass the barrier when presented in aqueous solutions adjusted to pH 7.6. This suggested that the different microspecies did take different routes through the skin at this pH and it accords with previous work, which has shown that ionic drug aggregates have the potential to pass the skin via as the transappendageal route [41]. It was thought to be problematic to study the transappendageal route for this molecule as tagging the drug with a marker to allow tracking through the skin would change its properties. However, this was not thought to significantly detract from the study results because despite the multiple routes of transport available for tetracaine and the possible involvement of the transappendial route at pH 7.6, passive transmembrane transport was still considered to be the dominant mode of barrier penetration for this molecule. This is why the drug aggregation, which presented the membrane with large supramolecular structures that needed to dissociate to provide free drug to penetrate the membrane, increased the lag-time of the transmembrane transport process [42], [43], [44].

## 4 Conclusions

The data from the current study demonstrated that the aggregation process of tetracaine molecules was more favourable in the aqueous vehicles at pH 7.6, 9 and 10 and this resulted in different supramolecular structures to be formed in solution when compared to the aqueous vehicles at pH 4 and 6. The type of supramolecular structure produced in solution seemed to have an influence on transmembrane penetration when applied to both porcine skin and a silicone membrane. Unionised tetracaine molecules had a higher affinity with the hydrophobic membranes and this generated the highest flux values but the process of transport was initially retarded by the aggregate formation. Spectroscopic analysis of the aggregates showed that the unionised microspecies promoted the formation of larger hydrophobic structures and it was the production of these structures which were suggested to lead to the delay in clinical action of the commercial tetracaine formulation when applied to the skin.
