# Supplementary material for: Investigating how the attributes of self-associated drug complexes influence the passive transport of molecules through biological membranes
Source: Eur J Pharm Biopharm. 2016 May;102:214–22. doi: 10.1016/j.ejpb.2016.03.002 (PMC4827376; doi:10.1016/j.ejpb.2016.03.002)
Supplement: Supplementary Fig. S1 and Tables S1–S4 [file mmc1.docx]

**Supplementary data**

**
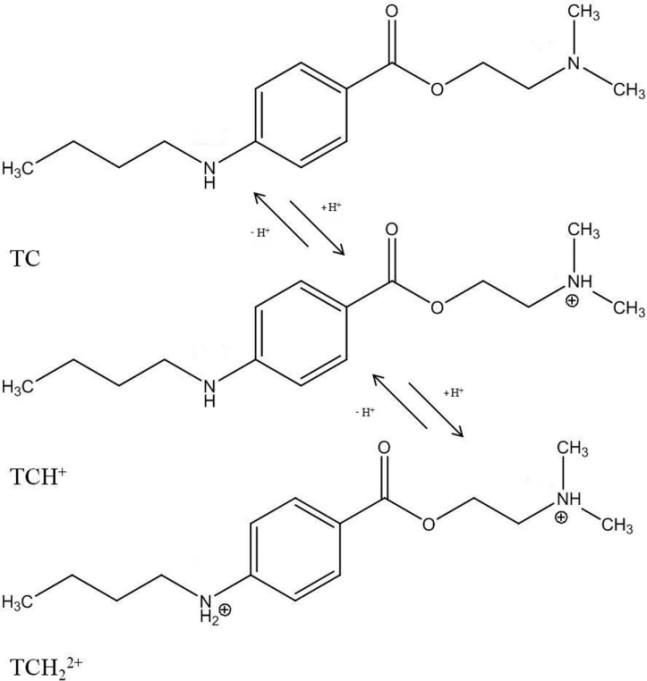
**

***Figure S1.*** Schematic representation of tetracaine ionization equilibrium, exhibiting two ionizable groups (pKa’s 2.48 ± 0.03 and 8.56 ± 0.02).

***Table S1*.** Percentage of the microspecies (TC, TCH^+^ and TCH_2_^+2^) in solution with an increasing pH (source: HYSS software).

| **pH** | **% TC** | **%TCH^+^** | **%TCH_2_^+2^** |
| --- | --- | --- | --- |
| 4 | 0.03 | 93.5 | 6.47 |
| 6 | 0.23 | 99.7 | 0.07 |
| 7.6 | 9.88 | 90.1 | 0.02 |
| 9 | 73.4 | 26.6 | negligible |
| 10 | 96.5 | 3.5 | negligible |

***Table S2*.** Tetracaine proton chemical shifts in D_2_O at 32 °C (ppm) at pH 4, 6, 7.6, 9 and 10. The peak assignment was obtained using ChemNMR (Perkin Elmer Ltd., UK). (s) singlet; (d) doublet; (t) triplet and (q) quartet.

|  | **pH 4** | **pH 6** | **pH 7.6** | **pH 9** | **pH 10** |
| --- | --- | --- | --- | --- | --- |
| H1 | 0.88 (t) | 0.88 (t) | 0.89 (t) | 0.89 (t) | 0.89 (t) |
| H2 | 1.32 (q) | 1.33 (q) | 1.33 (q) | 1.33 (q) | 1.33 (q) |
| H3 | 1.47 (q) | 1.48 (q) | 1.48 (q) | 1.48 (q) | 1.48 (q) |
| H4 | 2.92 (t) | 3.12 (t) | 3.17 (t) | 3.16 (t) | 3.15 (q) |
| H5 | 6.5 (d) | 6.5 (d) | 6.73 (d) | 6.74 (d) | 6.74 (q) |
| H6 | 7.68 (d) | 7.68 (d) | 7.85 (d) | 7.86 (d) | 7.86 (q) |
| H7 | 4.35 (t) | 4.3 (t) | 4.9 (t) | 5.56 (q) | 5.51 (t) |
| H8 | 3.46 (t) | 3.5 (t) | 3.57 (t) | 3.60 (t) | 3.86 (t) |
| H9 | 2.87 (s) | 2.86 (s) | 2.86 (s) | 2.87 (s) | 2.86 (s) |

***Table S3*.** Summary indicies for tetracaine transport studies through a synthetic silicone membrane. Data represents mean ± SD (*n* = 5). ***p* < 0.01, ****p* < 0.001 (One-way ANOVA with Tuckey’s HSD test).

| **pH** | **Steady-state flux**  **(µg.cm^-2^.min^-1^)** | **Lag time**  **(min)** | **Diffusion coefficient**  **(× 10^-3^ cm^-2^.min^-1^)** |
| --- | --- | --- | --- |
| 4 | 2.5 ± 0.3 | 3.1 ± 0.8 | 2 ± 0.8 |
| 6 | 11.1 ± 3^***^ | 5.2 ± 0.7^**^ | 1.6 ± 0.3 |
| 7.6 | 13.6 ± 2.1 | 1.3 ± 0.4^***^ | 5.3 ± 0.9^***^ |
| 9 | 14.6 ± 1.2 | 14.2 ± 1.1^***^ | 0.51 ± 0.04^***^ |
| 10 | 17.1 ± 3.3 | 13.6 ± 0.9 | 0.63 ± 0.08 |

***Table S4*.** Summary indicies for tetracaine transport studies through porcine skin. Data represents mean ± SD (*n* = 5). **p* < 0.05, ****p* < 0.001 (One-way ANOVA with Tuckey’s HSD test).

| **pH** | **Steady-state flux**  **(µg.cm^-2^.min^-1^)** | **Lag time**  **(min)** | **Diffusion coefficient**  **(× 10^-3^ cm^-2^.min^-1^)** |
| --- | --- | --- | --- |
| 4 | 0.96 ± 0.3 | 123 ± 3.9 | 0.016 ± 0.003 |
| 7.6 | 2.17 ± 0.2^***^ | 202 ± 6.7^***^ | 0.001 ± 0.0002^***^ |
| 9 | 1.59 ± 0.3^*^ | 243 ± 10.9^***^ | 0.008 ± 0.0001 |
